# Determinants of maternal health care utilization in Holeta town, central Ethiopia

**DOI:** 10.1186/1472-6963-13-256

**Published:** 2013-07-03

**Authors:** Kidist Birmeta, Yohannes Dibaba, Desalegn Woldeyohannes

**Affiliations:** 1 College of Development Studies, Center for Population Studies, Addis Ababa University, Addis Ababa, Ethiopia; 2 Department of Population and Family Health, College of Public Health & Medical Sciences, Jimma University, Jimma, Ethiopia; 3 Department of Public Health, School of Medicine and Health Sciences, Addis Ababa Science and Technology University, Addis Ababa, Ethiopia

**Keywords:** Antenatal care, Delivery care, Holeta town, Ethiopia

## Abstract

**Background:**

In developing countries a large number of women are dying due to factors related to pregnancy and child birth. Implementing and assuring utilization of maternal health care services is potentially one of the most effective health interventions for preventing maternal morbidity and mortality. However, in Ethiopia the utilization of maternal health care is low.

**Methods:**

A cross-sectional study was conducted from January 20 to February 20, 2012 in Holeta town, central Ethiopia, to assess the determinants of maternal health care utilization among women who had given birth in the past three years prior to the survey. Structured questionnaire and focus group discussion guides were used for data collection. Data were collected from a sample of 422 women in the town. Descriptive, bivariate and multivariate logistic regression analyses were conducted. Statistical tests were done at a level of significance of p < 0.05.

**Results:**

The study revealed that 87% of the women had at least one antenatal visit during their last pregnancy. Among the antenatal service users, 33.7% had less than four antenatal visits. More than half of the antenatal care (ANC) attendants made their first visit during their second and third trimester of pregnancy although WHO recommended ANC should be started at the first trimester of the pregnancy. There was a significant association (P<0.05) between ANC attendance and some demographic, socio-economic and health related factors (age at last birth, literacy status of women, average monthly family income, media exposure, attitude towards pregnancy, knowledge on danger signs of pregnancy and presence of husband approval on ANC). The study also revealed that about 61.6% of the women had given birth in the health institutions. Parity, literacy status of women, average monthly family income, media exposure, decision where to give birth, perception of distance to health institutions (HI) and ANC attendance were found to be significantly associated (P<0.05) with delivery care (DC) attendance.

**Conclusions:**

The utilization of ANC and DC service is inadequate in the town. The utilization of ANC and DC were influenced by demographic, socio-economic and health related factors. Improving the status of women by expanding educational opportunities, strengthening promotion of antenatal and delivery care by enhancing community awareness about the importance of ANC and DC are recommended.

## Background

Globally, an estimated 287,000 maternal deaths occurred in 2010. Developing countries accounted for 99% (284,000) of deaths. Among the developing regions, sub-Saharan Africa had the highest maternal mortality ratio (MMR) at 640 per 100,000 live births [1]. Maternal and neonatal morbidity and mortality rates in Ethiopia are among the highest in the World. According to World Health Organization (WHO) 2012 report, 9000 maternal deaths occurred in Ethiopia in 2010 [1]. The 2011 Ethiopian Demographic and Health Survey (EDHS) showed that the maternal mortality ratio was 676 deaths per 100,000 live births. In other words, for every 1,000 live births, about seven women (6.76) died during pregnancy, childbirth, or within two months of childbirth. The neonatal mortality rate of Ethiopia in 2011 was 37 deaths per 1,000 live births [2].

Maternal health care (MHC) service is potentially one of the most effective health interventions for preventing maternal morbidity and mortality in particular in places where the general health status of women is low. In addition, maternal health care gives opportunities for delivering health information and services that can significantly promote the health of the women and their infants. But in developing countries like Ethiopia the potential to deliver health information and services using MHC still remains underutilized.

In Ethiopia, one explanation for poor health outcomes among women is the nonuse of modern health care service by a great proportion of women in the country [3]. According to the 2011 EDHS report, the coverage of antenatal care was 34%. This varied from 76% for women residing in urban areas to 26% of women in rural areas. Even among those who use ANC, lower proportions of women receive care according to WHO recommendations (beginning ANC in the first trimester, and making four antenatal care visits). One woman in every five (19 percent) made four or more antenatal care visits during the course of her pregnancy and the median duration of pregnancy at the time of the first antenatal visit is 5 months [2]. Moreover, a large majority of the births (90%) in Ethiopia occur at home, and only 10% of births are delivered with the assistance of a trained health professional [2].

Hence considering global and national interests in the Millennium Development Goal and Ethiopia’s high level of maternal mortality, understanding the factors that determine maternal health care utilization is crucial. The result of this study identifies the main determinants of the utilization of maternity care services in Holeta town. If effective implementation is done to avoid the investigated determinants to utilization of MHC services it benefits the society, particularly women and children to have a better utilization of MHC services which will improve their health status and wellbeing. The findings also help governmental and non-governmental organizations in planning and implementing programs to reduce maternal morbidity and mortality in the study area. Therefore, the purpose of this study is to assess the factors that determine utilization of maternal health care- antenatal care (ANC) and delivery care (DC) - services in Holeta town, central Ethiopia.

## Methods

### Study design and setting

A cross-sectional study that employed both qualitative and quantitative data collection methods was carried out to assess factors determining utilization of maternal health care services. The study was conducted from January to February, 2012. Holeta town is located at about 29 kms from Addis Ababa (the capital) in the western direction. Administratively, the town is subdivided in to eight *kebele*s^a^. Majority of the residents of the town depend on agriculture and petty trade for their livelihoods. Based on the information obtained from woreda health office, in 2011 the population of Holeta town was about 37,850 of which half (50.4%) were females. The population of Holeta obtains health services from one government owned health center, seven health posts, two medium private clinics, ten small clinics and one special private clinic. Most of these facilities run integrated maternal health services routinely staffed with senior and junior maternal health care providers.

### Sample size and sampling procedures

The study subjects were a sample of women in the child bearing age, who had given birth in the past three years prior to the survey and residents of the town. A sample size of 422 was determined using the formula for single population proportion based on the assumption**s:** in the absence of the previous prevalence data on the population under study, and to obtain the maximum sample size, p was assumed to be 0.5. Moreover, a margin of error of 5%, a confidence interval of 95% assumed (Z_a/2_=1.96), and 10% contingency for non-response were used to calculate a sample size of 422 women.

Then, a multi-stage sampling scheme was used to identify the study subjects. From a total of eight *kebele*s in the town, four *kebeles* were randomly selected by using a lottery method. Secondly, the number of households living in the area was recorded; the located sample size for each *kebele*s was obtained using probability proportional to the size of households found in each *kebele*. From the four sampled *kebeles*, a total of 422 study samples were selected using systematic sampling procedure. For households with more than one eligible woman, interview was done by selecting a women using lottery method, although in the event of a household with no eligible woman the immediate next household was interviewed. Revisits of two times were made in case where eligible respondents were not available at the time of the survey. Regarding a women having two under three children the most recent birth was taken.

The study also uses qualitative data from focus group discussions (FGDs). Semi structured open ended and non directive (FGD) guide was designed in order to triangulate responses obtained by the structured questionnaire on the knowledge of maternal health care utilization (specifically ANC and DC) knowledge of danger signs of pregnancy and delivery, primary reasons for ANC non- attendance, and type of assistances during delivery) advantage and disadvantage of home delivery. For the qualitative study, non probabilistic purposive sampling technique was used. Each FGD consisted of eight women.

Data were collected by eight female data collectors who had a minimum of secondary level education. They had two days of training for the purposes of data collection. The data collection process was closely supervised by field supervisors and the research team.

The study obtained ethical approval from the Center of Population Studies, Addis Ababa University. The survey commenced after written consent obtained from Holeta health bureau and district council. Informed verbal consent was requested from each study subjects. Each respondent were informed about the objectives of the study and assurance of confidentiality.

### Data analysis

After the data collection is completed and questionnaires edited and coded, the data were entered and processed by using SPSS version 17. Descriptive analysis like percentage, mean, standard deviations were used to describe the study population in relation to demographic and socio-economic and other relevant variables. Bivariate and multivariate logistic regression analyses were done to identify the association between the independent and outcome variables. The chi-square test was used to identify independent variables, which have association with the dependent variable that would be retained for further analysis at the multivariate stage. Further, multivariate analysis carried out to explore the net effect (relative risk) of all independent variables on the dependent variable by controlling possible intervening variables. Antenatal care was defined as if the women had received antenatal care check-up at least once during their pregnancy from formal sources. Similarly, delivery care defined as if the delivery took place at formal public and private facilities by health professionals. For the qualitative study, thematic content analysis was done.

## Result

### Socio-demographic profile

A total of 422 women who had given birth in the past three years prior to the survey were interviewed. From all eligible women in the selected samples, 419 women responded to the questionnaire which made the response rate to be 99.2%. Most of the study participants were in the age group of 20–34 (79.5%), with a mean age of 27.4 ± (6.1) years. More than half (68%) of the respondents have attended school; whereas about 32% of the respondents have never attended school. Regarding occupation, most women (57.3%) were housewives (Table 1).

**Table 1:** Selected percentage distributions of general characteristics of respondents in Holeta town January to February, 2012, (N =419)

**Variables**		**Frequency**	**Percentage**
**Age**	15-19 years	16	3.8
	20-34 years	333	79.5
	35-49 years	70	16.7
	Mean ±SD		(27.4 ± 6.1)
**Literacy status**	No schooling	134	32
	Schooling	285	68
**Occupation**	House wife	240	57.3
	Other*	179	42.7
**Average monthly family income**	below 401	134	31.7
	401-1000	174	41.8
	above 1000	111	26.5
	Mean ±SD		(797.3 ±669.9)
**Family size**	1-3	123	29.4
	4-5	203	48.4
	above 5	93	22.2
	Mean ±SD		(4.6±1.7)
**Media exposure**	No exposure	122	29.1
	Radio or TV	150	35.8
	Radio and TV	147	35.1

* Students, daily laborer, civil servant, merchant, maid servant, etc.

1 USD= 17.50 Ethiopian currency at the time of survey (2012).

### Maternal health care utilization

Out of all the respondents included in the study, 87.1% had at least one antenatal visit during their last pregnancy. Nearly 42% of the women made their first antenatal visit in their first trimester of pregnancy, while majority (54.5%) of women had their first antenatal visit in their second trimester. Among the antenatal service users 33.7% had less than four antenatal visits during their last pregnancy.

**Figure 1:**
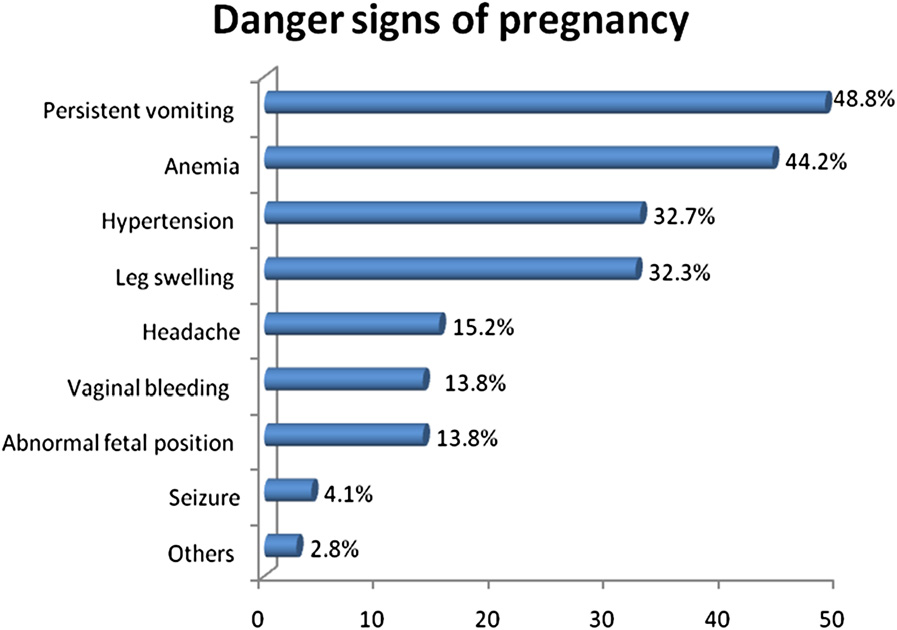
Percentage distribution of respondents’ knowledge about Danger Signs of Pregnancy in Holeta town, January to February 2012, (n=217).

Concerning place of last delivery, 38.4% of the deliveries took place at home and 61.6% at health institutions. Among the home deliveries 96% were attended by TTBAs, untrained TBAs, relatives and/or neighbors. Knowledge of danger signs of pregnancy was also assessed. Accordingly 48.8% of respondent reported persistent vomiting, 44.2% report anemia, 32.7% hypertension (Figure 1).

### Main reasons for ANC non-attendance

**Figure 2:**
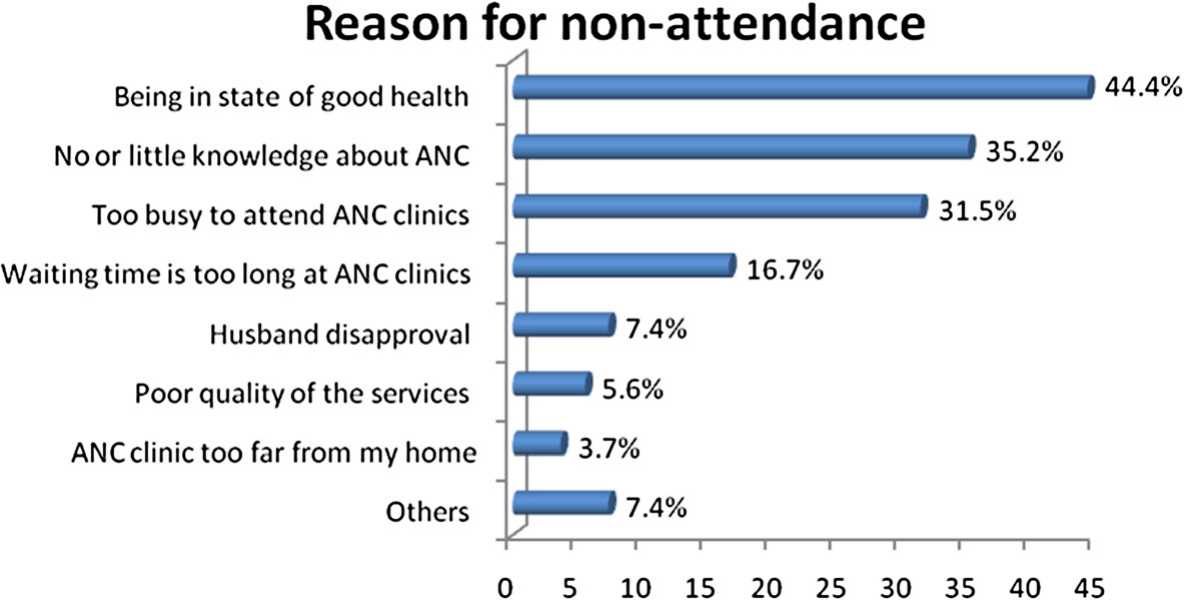
Percentage distribution of Reasons for non Attendance ANC Holeta Town, January to February, 2012 (n=54).

Among the 54 women did not ANC the reported reason for non attendance includes absence of illness (44.4%), no or little knowledge about ANC (35.2%), and being too busy 31.5% (Figure 2).

Also in the FGDs the majority of participants agreed the main reasons for not attending ANC were lack of awareness, apparently being healthy, work overload either in the household or in the other daily activities, long waiting time, financial constraint, and confidence on local traditional birth attendants (TBAs).

One of the participants from FGD1 aged 29, married and parity of three said*,” some of the women do not want to go to health institutions for fear of human immune deficient virus (HIV) test during ANC visit. If the result is positive other people may know about them being HIV positive, not knowing the HIV test during pregnancy is voluntary and confidential”.*

### Respondents perception about the quality of the ANC service and health service

#### Factors

Out of the 365 ANC users asked about the personal respect of health workers at ANC unit, the majority 91.2% reported that health workers were respectful for them. As to waiting time for ANC services, 52.9% said they waited short time, 38.6% and 8.5% said they waited fair time and long time, respectively. Women were also asked about a lack of privacy at ANC unit and 81.6% of them reported there was no lack of privacy and 42(11.5%) of the women reported that there was a problem of privacy. 189(51.8) report good, 131(35.9%) report satisfactory and 45(12.3%) report poor quality of services.

Regarding the quality of the ANC services, participants of FGDs stated that some health workers were not treating the women politely, especially during delivery care. But in general, the health care providers in their area are respectful. Most of the women in both FGDs agreed that over all their perception on quality is good but there are some problems associated with inadequate skilled professionals, manner of some health providers, and shortage of equipments and sometimes there is lack of privacy. One of the discussant stated that, “*If you go to health institution you will find low quality of services and lack of respect and mistreatment from some of the health care providers*”.

#### Reasons for preferring home delivery

**Figure 3:**
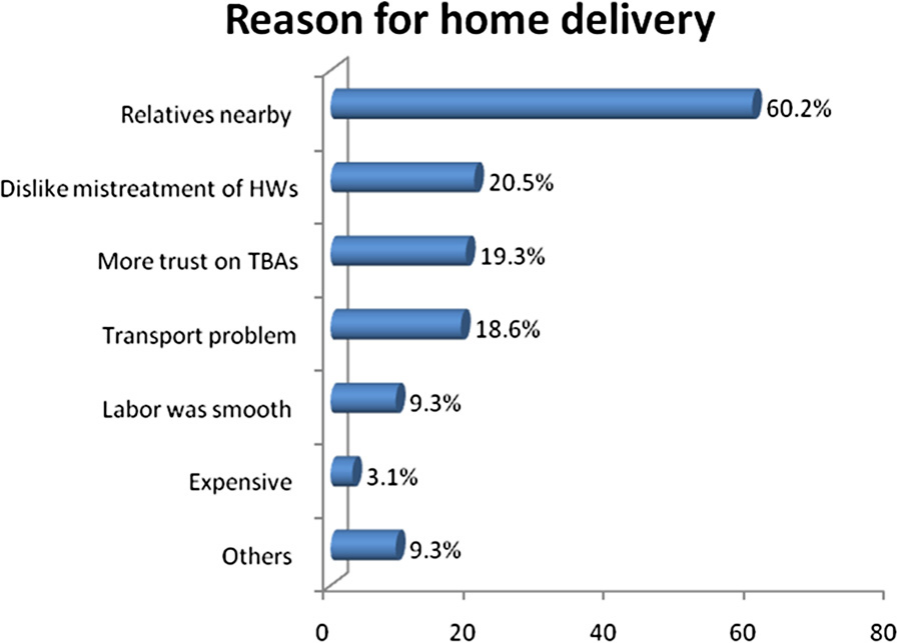
Percentage distribution of Reason for Preferring Home Delivery in Holeta Town, January to February, 2012 (n=161).

Among 161 women who delivered at home 97 (60.2%) reported that they prefer to deliver at home where close relatives are nearby than Health Institutions, 20.5% reported that they prefer to give birth at home because they dislike mistreatment by health workers, 19.3% reported they have more trust on TBAs than health professionals (Figure 3).

In the FGDs, although there were differences in opinion of home delivery and health institutional delivery, most women agreed that health institutional delivery is best for both the mother and child’s health.

One FGD participant said *“some women attend antenatal care throughout the whole pregnancy period but usually they deliver at home because labour is unpredictable and arises suddenly without warning and especially if it is in the night time due to transport problem the women do not have much choice rather than delivering at home”.*

Another discussant said, “*Most women with experience in giving births more than once did not want to go to health institutions because they think that they had enough experience and don’t need assistance of health workers”.* Another participant from the FGD2 aged 28 and parity of two added that “Mostly *the health workers giving delivery care services in the health institution are students and do not have adequate knowledge to assist child delivery because of this fear some women rather prefer to give birth at home with the assistance of TBAs*”.

#### Determinants of ANC utilization

The multivariate analysis for ANC revealed that the odds of attending ANC is 1.2 times higher (OR=1.168) for women in the age group of 20–34 as compared to those in the age group 15–19 women. Women with some education were more than two times more likely to attend ANC (OR=2.645) as compared with those who had no education (Table 2). The multivariate analysis result also showed that the likelihood of utilizing ANC decreased, as the family income gets lower. From the Table 2, it may be observed that respondents who had an average monthly family income of less than 23 USD (401 ETB) were less likely to attend ANC as compared with those whose average monthly family income of above 57 USD (1000 ETB).

**Table 2:** Multivariate logistic regression analysis results of respondents in ANC attendance, in Holeta town, January to February, 2012, (n=419)

**Variables**	**β**	**Sig(p)**	**Exp (β) (OR)**	**95% C.I. for EXP(β)**	
				**Lower**	**Upper**
**Age at last birth**					
15 – 19^**(RC)**^					
20 – 34	.155	.804	1.168	.343	3.976
35 - 49	-.700	.040	.183*	.036	.927
**Parity**					
Parity 1	-.566	.448	.568	0.132	2.448
Parity 2-4	.021	.973	1.021	0.304	3.430
Parity 5 and above ^**(RC)**^					
**Literacy status**					
No Schooling ^**(RC)**^					
Schooling	.973	.034	2.645*	1.078	6.487
**Occupation of the respondent**					
House wife	.593	.171	1.809	0.775	4.222
Others (civil servant students, maid servant, merchant etc…) ^**(RC)**^					
**Marital status**					
Married	.058	.907	1.067	0.398	2.821
Others( divorced, widowed, never married)^**(RC)**^					
**Average family monthly income**					
Below 23 USD^1^	−1.922	.034	.146*	.025	.867
23 - 57 USD	−1.401	.124	.246	.041	1.470
Above 57 USD^**(RC)**^					
**Media exposure**					
No exposure ^**(RC)**^					
Radio or TV	1.389	.006	4.013**	1.487	10.831
Radio and TV	1.485	.037	4.416*	1.097	17.784
**Planned pregnancy**					
Yes ^**(RC)**^					
No	−1.087	.021	.337*	0.134	0.850
**Knowledge on danger signs of pregnancy**					
Better knowledge	1.264	.019	3.541*	1.236	10.147
Poor knowledge ^**(RC)**^					
**Presence of husband approval on ANC**					
Yes	2.197	.000	8.999***	3.706	21.855
No ^**(RC)**^					

1 USD= 17.50 Ethiopian currency at the time of survey (2012).

* = P < 0.05 ** = P < 0.01 *** = P < 0.001.

RC = Reference category.

Similarly, media exposure has association with the ANC attendance. Women who had exposure to radio or television (TV) were four times higher attended ANC than those with no exposure (OR=4.013). Regarding attitude to recent pregnancy, women who reported that they did not plan the pregnancy were 67% less to attend ANC than those who report they planed the pregnancy (OR=0.337).

The odds of utilizing ANC were more than three times likely for those with better knowledge of danger signs of pregnancy than those with poor knowledge (OR=3.541). Similarly, the utilization of ANC was almost nine times more likely for women reported their husbands approve ANC than women with those whose husbands did not approve ANC service (OR=8.99) (Table 2).

#### Determinants of delivery care utilization

Women with parity of one are nearly three times (OR= 2.845) as likely as women of parity five and above to attend delivery care. Literacy status has positive association with delivery care utilization. The multivariate analysis for DC revealed that respondents with education were more than three times likely to use to give birth at health institutions than uneducated women (OR= 3.861). With regards to income, women lower family income are less likely to use delivery care (OR= 0.372) as compared to women with monthly higher family income (Table 3). Women who had exposure to Radio or TV were almost three times more likely to attend DC than those with no exposure to media (OR= 2.998). And those who reported that they had exposure to radio and TV attend DC two times more than those with no exposure (OR= 2.146).

**Table 3:** Multivariate logistic regression analysis results of respondents DC in Holetatown, January to February, 2012 (n=419)

**Variables**	**β**	**Sig (p)**	**Exp (β) (OR)**	**95% C.I. for EXP(β)**	
				**Lower**	**Upper**
**Age at last birth**					
15 – 19^**(RC)**^					
20 – 34	-.652	.144	.535	.232	1.237
35 –49	-.854	.618	.426	.127	1.431
**Parity**					
Parity 1	1.046	.044	2.845*	1.026	7.886
Parity 2-4	.799	.090	2.223	0.884	5.593
Parity 5 and above ^**(RC)**^					
**Literacy status**					
No Schooling ^**(RC)**^					
Schooling	1.351	.000	3.861***	2.165	6.883
**Occupation of the respondent**					
House wife Others (civil servant students, maid servant, merchant etc…) ^**(RC)**^	-.069	.809	.934	0.535	1.628
**Marital status**					
Married	.695	.052	2.004	0.995	4.036
Others( divorced, widowed, never married)^**(RC)**^					
**Average family monthly income**					
Below 23 USD^1^		.014	.372*	.168	.821
23-57 USD		.490	.778	.382	1.587
Above 57 USD^**(RC)**^					
**Media exposure**					
No exposure ^**(RC)**^					
Radio or TV	.098	.002	2.998**	1.504	5.975
Radio and TV	.764	.034	2.146*	1.058	4.351
**Decision where to give birth**					
Self/jointly with husband	.961	.002	2.615**	1.421	4.814
Others (husband or other familymembers) ^**(RC)**^					
				
**Perception of distance to HI**					
Very close^**(RC)**^					
Average	-.736	.015	.479*	0.264	0.868
Too far	.330	.602	1.391	0.403	4.808
**ANC attendance**					
Yes^**(RC)**^					
No	−2.000	.001	.135**	.042	.438

^1^ USD= 17.50 Ethiopian currency at the time of survey (2012).

* = P < 0.05 ** = P < 0.01 *** = P < 0.001.

RC = Reference category

The result of the multivariate analysis also revealed that the likelihood of attending DC among those women the decision where to attend DC was made by themselves or jointly with their husbands attended DC two times more than those women where decision were made by their husband or others family members (OR= 2.146). Women who did not attend ANC service were 86.5% less likely to attend DC than women who were ANC attendants (OR= 0.135) (Table 3).

## Discussion

The study assessed factors that determine the utilization of maternal healthcare services in Holeta town. The revealed that 87.1% women sought at least one ANC from modern health care providers. However, a considerable number do not make the minimal number of visits (four) as recommended by the WHO. The finding of this study is comparable with findings of studies conducted in Jimma town (90%), in Jijiga town (82%) and in Hadiya zone **(**86%) respectively [4-6]. According to the 2011 Ethiopian Demographic and Health survey (EDHS), 76% of women in urban areas used ANC [2]. The comparative figures for rural areas and national level was 26% and 34% respectively. Holeta town has higher level of ANC use may be due to the fact that the town is near to the capital city of Ethiopia and could have better opportunities for information and better access to health institutions than the other urban areas. The other explanation could be this study considers women who gave birth three years prior to the survey and the recently started urban health extension program (HEP) is providing equitable access to promotive, preventive and select curative health interventions through health extension workers (HEWs). Among the services based on health packages, maternal health is part of the family health package.

In the study, the primary reasons given for not attending ANC services include being in a state of good health, no or little knowledge about ANC, being too busy, too long waiting time, poor quality of services, husband disapproval and far distance from home to health services etc. Other studies also reported similar reasons [7-9].

Antenatal care is more effective in preventing adverse pregnancy outcomes when it is sought early in pregnancy and is continued throughout pregnancy [10]. More than half (58%) of women in this study area made their first antenatal visits in their second and third trimester of pregnancy. This indicates that, a considerable number of women in the study area start ANC at relatively late stage of pregnancy.

In the FGDs, women pointed out that the barriers to the utilization of antenatal care services were lack of awareness, apparently being healthy, work overload either in the household or in the other daily activities, long waiting time, financial constraint, and confidence on local TBAs. According to FGDs participants, some women did not attend ANC due to misconception on HIV test during ANC not knowing it is voluntary and confidential.

Home delivery is still a norm in many parts of Ethiopia. In this study, 38.4% of births had taken place at home. This finding is consistent with the findings of the studies in other towns in Ethiopia [6,7,11]. According to the 2011 EDHS, nearly half of births taken place at home in urban areas [2]. This difference of institutional delivery could be explained by the fact that the present study area was done near to the capital city of Ethiopia where women tend to have better access to health facilities, education, and information about maternal health care service.

Delivery care is an important component of efforts to reduce the health risks of mothers and children and increase the proportion of babies delivered under the supervision of health professionals in different health institution [12]. According to women on both FGDs groups, the main barriers that affect the utilization of delivery care services are desire to deliver where their relatives in their surrounding when giving birth, bad experience with the health care system, unexpected delivery, transport problem and financial constraint. The FGDs participants also indicated that some women with repeated experience of child birth did not want to go to health institution because of their previous experience which often led them to believe that they do not need assistance from the health professional.

Our multivariate analysis showed that age, education, income, exposure to media, and presences of husband approval were significantly associated with ANC utilization. For instance, when considering age, it was found that women in the age group 35–49 were less likely to use ANC service than women in the age group of 15–19. Several studies found out that women’s age plays a significant role in the utilization of maternal health care [9,13,14]. This might be due to the fact that younger women are more cautious about their pregnancies and sought trained professionals but older women tend to believe that modern health care is not necessary due to experiences and accumulated knowledge from previous pregnancies and births.

The findings of this study revealed that education is strong predictor of maternal health care utilization for both ANC and DC services. The result is similar with other results which revealed that education has a positive relationship with maternal health care utilization [2,5,11,15]. It is because that educated mothers are considered to have a greater awareness of the existence of maternal health care services and benefited in using such services. They are most likely to have better knowledge and information on modern medical treatment. Education is an opportunity to empower women; and empowered women have greater confidence and capability to make decision to use modern health care services for themselves and for their children.

Quality is also important in maternal health programmes and can increase the likelihood that women facing obstetric emergencies will go to health facilities for life-saving care [16]. These factors will act as inhibitors of future utilization, thus affecting the decision to seek care. As most of the FGDs discussant indicated in their opinion, there is a problem in the quality of care with some health institutes with low quality and mistreatment from some health professionals, which made some women not to seek the service on both ANC and DC services.

Husband’s or partner’s approval of ANC was most significantly related to antenatal care attendance. The result of the present study is found to be similar with previous studies in Ethiopia [9,17,18]. A study in Addis Ababa showed the risk of non-attendance was high for pregnant women whose husband’s attitude was negative or unknown [13]. It is expected that having a husband who approves antenatal care significantly increase the likelihood that a woman used antenatal care, irrespective of the husband’s background characteristics. Therefore, efforts to improve husband’s or partner’s attitude would probably increase utilization of health services by women.

Women’s attitude towards their current pregnancy, i.e. whether or not the pregnancy was planned, was found to affect ANC utilization. A woman’s attitude towards her pregnancy and the presence of social support has been found to influence ANC use in developing countries [19]. The study in Yirgalem and Jimma also revealed the same result [18]. The uses of ANC were significantly more among women with planned pregnancies [13]. Unplanned pregnancy is highly suggestive of lack of access to appropriate family planning opportunities. ANC might be an appropriate point of contact for promotion of family planning.

Delivery care utilization may also be affected by the person(s) who make the decision on the place where to give birth. From the result of this study, women for whom the decision on place of birth made by themselves or jointly with their husbands were two times more likely to utilize DC than women whom decision where to give birth made by others. Women, who had low decision-making power, result from lack of access to and control over economic resources [19]. This could be due to the high cost associated with pregnancy-related healthcare services in private health sector and/or women’s low autonomy to make decision in the household. This study is consistent with the result findings of other study [17].

Antenatal care visits had significant positive relation with utilization of DC services. In this study women who were non attendants of ANC were 86.5% less likely to utilize DC than ANC attendants. This is due to the fact that during antenatal care women are provided with information about the necessary follow-ups during pregnancy and after delivery and where to give birth at delivery. The study shows similar result with other studies [5,7,8,17].

### Limitations of the study

Despite the contribution of the study to the literature on maternal health care, this study has some limitations. First, it is a cross-sectional study in which temporal relations could not be assessed. There could be recall bias since the women were asked for events within the last three years prior to the survey despite the fact that, the most recent births were considered.

## Conclusions

This study demonstrated that utilization of maternal health services (antenatal and delivery services) during the period of three year preceding the survey were relatively higher in the study area but inadequate in general. The majority of women sought at least one antenatal visit from modern health care providers during their recent pregnancy. Most of ANC attendants visit ANC services after the first trimester. And a considerable number of women had less than four visits during their recent pregnancy which is against the recommendation by WHO which stated a minimum level of care to be four visits throughout the pregnancy [20].

Almost all of home deliveries were attended by TBAs (TBA, close relatives/ friends, neighbors and TTBA). The main reasons given by the individual for home delivery by most women were the presence of relatives nearby at home than health institutions, being healthy, more trust on TBA.

This study shows that the most important factors influencing the use of maternal health services in the study area are demographic, socio-economic and health related factors in nature. However, this does not detract from the relevance of service-related factors, especially. The demographic and socioeconomic factors identified in this study include education, age, income, exposure to media, and presences of husband approval and decision on the place where to give birth.

Improving educational opportunity for women may have a large positive impact on improving utilization of ANC and DC services. The results of this study also highlight the importance of taking into account a husband’s attitudes when designing interventions intended to raise the use of formal prenatal-care services. And community education about pregnancy, child birth and postpartum, particularly, the danger signs of pregnancy, labour and delivery and the actions ensuing complication need to get particular attention.

## Endnote

^a^Smallest administrative unit in Ethiopia.

## Abbreviations

ANC: Antenatal care; CI: Confidence interval; DC: Delivery care; EDHS: Ethiopian demographic and health survey; FGD: Focus group discussions; HEP: Health extension program; HEWs: Health extension workers; HI: Health institution; HIV: Human immunodeficiency virus; MHC: Maternal health care; MMR: Maternal mortality ratio; OR: Odd ratio; TBA: Traditional birth attendant; TTBA: Trained traditional birth attendant; WHO: World health organization.

## Competing interests

The authors declare that they have no competing interests.

## Authors’ contributions

KB was responsible for the conception, design, data collection, data analysis, interpretation, and write-up and in the preparation of the draft manuscript. YD and DW were involved in the design, data analysis, interpretation, write-up and revision of the paper. All authors read the final manuscript.

## Pre-publication history

The pre-publication history for this paper can be accessed here:

http://www.biomedcentral.com/1472-6963/13/256/prepub
